# Thalamic Activation Modulates the Responses of Neurons in Rat Primary Auditory Cortex: An In Vivo Intracellular Recording Study

**DOI:** 10.1371/journal.pone.0034837

**Published:** 2012-04-13

**Authors:** Lei Han, Yonghai Zhang, Yunxiao Lou, Ying Xiong

**Affiliations:** 1 Department of Physiology, Chongqing Key Laboratory of Neurobiology, Third Military Medical University, Chongqing, People's Republic of China; 2 Department of Neurobiology, Chongqing Key Laboratory of Neurobiology, Third Military Medical University, Chongqing, People's Republic of China; University of Southern California, United States of America

## Abstract

Auditory cortical plasticity can be induced through various approaches. The medial geniculate body (MGB) of the auditory thalamus gates the ascending auditory inputs to the cortex. The thalamocortical system has been proposed to play a critical role in the responses of the auditory cortex (AC). In the present study, we investigated the cellular mechanism of the cortical activity, adopting an *in vivo* intracellular recording technique, recording from the primary auditory cortex (AI) while presenting an acoustic stimulus to the rat and electrically stimulating its MGB. We found that low-frequency stimuli enhanced the amplitudes of sound-evoked excitatory postsynaptic potentials (EPSPs) in AI neurons, whereas high-frequency stimuli depressed these auditory responses. The degree of this modulation depended on the intensities of the train stimuli as well as the intervals between the electrical stimulations and their paired sound stimulations. These findings may have implications regarding the basic mechanisms of MGB activation of auditory cortical plasticity and cortical signal processing.

## Introduction

Sound information travels from the peripheral organs to the auditory cortex (AC) through the auditory nuclei and pathways in the central auditory system. The auditory thalamus consists of the MGBv of the lemniscal pathway as well as the medial and dorsal nuclei of the nonlemniscal pathway [1]. The neurons in the MGBv are highly frequency-specific, exhibiting tonotopic organization. Electrical stimulation (ES) in the MGBv can elicit changes in the AC that adjust auditory signal processing in the frequency domain [2]. During early development, the arrival of thalamocortical input is fundamental for the development of cortical functional maps [3]. The thalamus plays a crucial role in adult animals in controlling the cortical state and modulating their responses [4]. Because the auditory thalamocortical pathway is the only neural substrate that sends precise frequency information to the AC, this system likely plays a critical role in auditory cortical responses, as is evidenced in various studies [3], [5], [6].

Our understanding of the thalamocortical contribution to cortical information processing is mostly based on studies in thalamocortical slice preparations [7]–[10]. It has been confirmed that the experimental induction of long-term potentiation (LTP) typically employs a high-frequency stimulation protocol, whereas long-term depression (LTD) is typically induced by a low-frequency stimulation protocol [11], [12]. Furthermore, the thalamocortical synapses also exhibit adaptation during periodic stimulations that can affect the magnitudes of cortical responses, which is called short-term depression (STD) [13]. These mechanisms all relate to the modifications of the cortical responses and sensory-specific receptive fields (RFs). However, the mechanism by which the electrical stimulation of the thalamus can affect sensory-evoked responses and signal processing in the sensory cortex is currently unknown [14].

Previous *in vitro* studies did not refer to adaptive changes in acoustic responses in the AC. Moreover, *in vivo* extracellular studies show that the focal ES of the ventral division of the medial geniculate body (MGBv) shifts the neuronal RFs of the primary auditory cortex (AI) toward the RFs of the stimulated MGBv neurons [2], [15]. Therefore, the thalamocortical system plays a critical role in the frequency-specific plasticity of the AC. A few studies using *in vivo* intracellular recording or patch clamping demonstrate that the neocortical responses and RFs can be modified by facilitating the synaptic transmission of the thalamocortical system through the electrical activation of the MGB [16], [17]. Recently, evidence was presented that different interactions between synaptic inputs within the cortex constitute the structural foundation of diverse cortical responses [18]. However, the intracellular mechanisms underlying the modulating effects of presynaptic (MGB) activation on postsynaptic (AC) auditory responses remain unclear.

**Figure 1:**
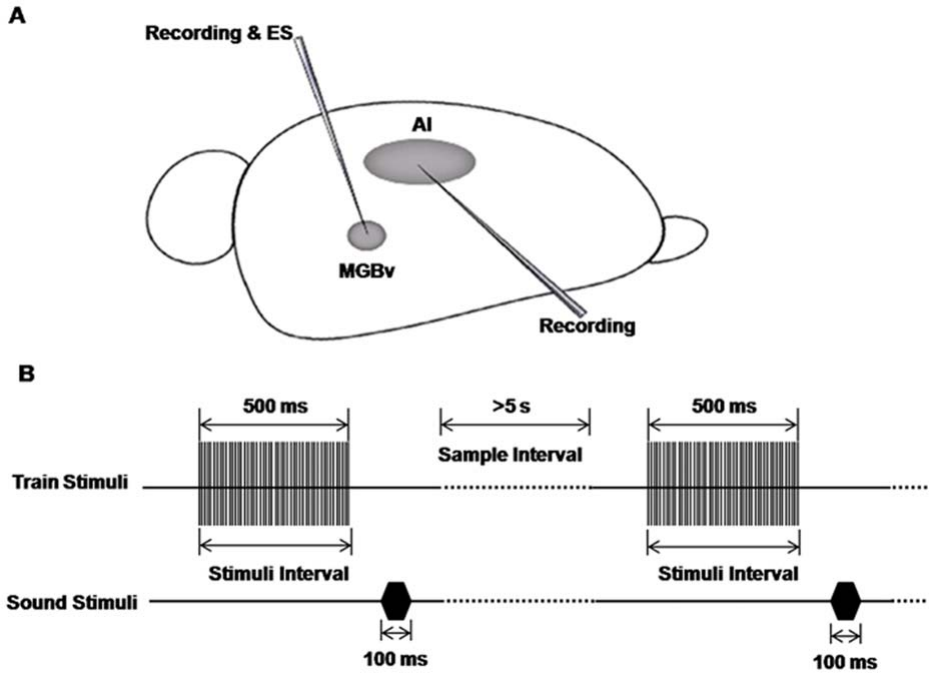
Schematic diagram of experimental configuration and procedure. **A**) A tungsten electrode was impelled into the right MGBv to record the electrophysiological characteristics of the neurons in the MGB and verify that the stimulating site was fully within the MGBv. Next, the tungsten electrode was fixed in place to stimulate the MGBv. After that, a sharp glass electrode was impelled to record the intracellular signals of AI neurons. **B**) Stimulation models. One sample consisted of an electrical stimulus of the MGBv paired with a testing white noise. The intervals between the onsets of the two stimuli were varied from 500 ms to 3000 ms. The intervals between the different samples were longer than 5 s.

In the present study, we examined how high- and low-frequency ES in the auditory thalamus affect cortical responses and signal processing in thalamocortical circuits. We monitored the changes in sound-evoked responses in the AC immediately after the high- and low-frequency electrical stimulation of the MGBv using intracellular recording to explore the effects of the ES on the cortical capabilities of response, encoding and processing of acoustic stimuli (Fig. 1). We found that low-frequency stimulation of the MGBv enhanced the amplitudes of sound-evoked excitatory postsynaptic potentials (EPSPs), whereas high-frequency stimulation depressed the cortical EPSPs in AI neurons.

## Results

**Figure 2:**
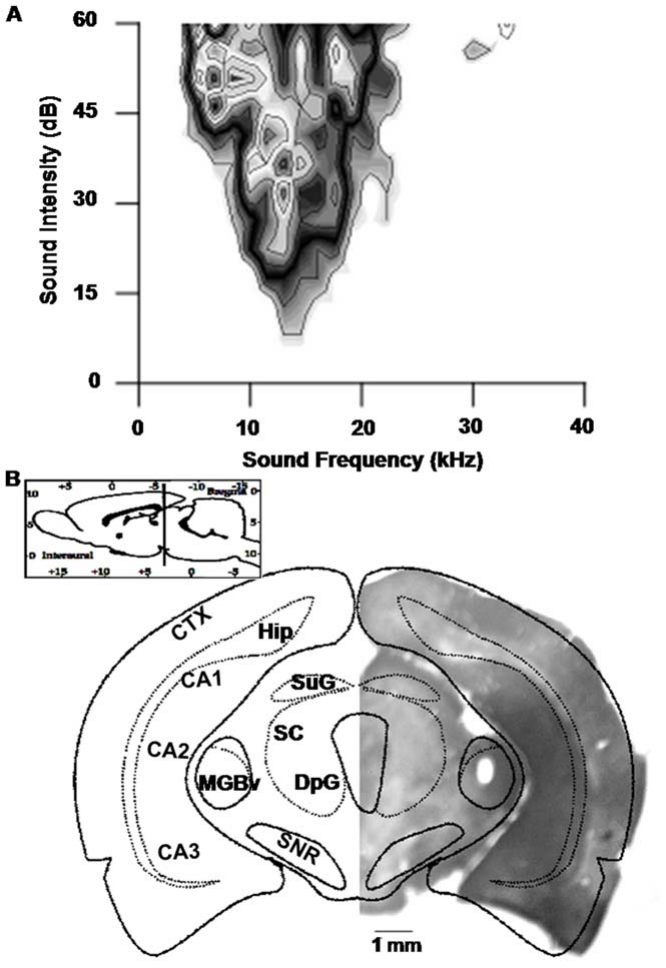
Confirmation of the location of the MGBv. **A**) The receptive field of the recorded MGBv neurons. This field had a typical “V" shape that was in concordance with the representative receptive field of the neurons in the MGBv, which further confirmed the location of the MGBv. The best frequency of this single unit was approximately 13 kHz, and its minimum threshold was approximately 7 dB SPL. **B**) The anatomical location of the site of electrical stimulation. A micrograph of the coronal section of the right brain compared with the atlas at 5.5 mm posterior to the bregma. A lesion site enwrapped with a thick layer of injured neurons could be observed at the corresponding position of the MGBv according to the atlas, whose boundary was sleek and clear-cut. Inset, position of the section in the rat brain. CTX, cortex; MGBv, ventral portion of the medial geniculate body; Hip, hippocampus; CA1, CA2, CA3, fields CA1, CA2, and CA3 of the hippocampus; SNR, reticular part of the substantia nigra; SC, superior colliculus; DpG, deep gray layer of the superior colliculus.

All the recorded MGBv neurons were sharply tuned to specific frequencies (Fig. 2A). The neurons were often recorded at a depth of 4500–5500 µm below the brain surface (Fig. 2B). Their best frequencies (BFs) ranged from 8 to 39 kHz, and their minimum thresholds (MTs) ranged from 5.6 to 30.4 dB SPL. These neurons were tuned to the frequencies within the central hearing range of rats.

Forty neurons were sampled from the AIs of 36 animals. The recording duration of a single neuron ranged from 20 min. to 360 min., with an average of 67.5±78.5 min. The depth of our recorded neurons ranged from 326 to 1256 µm below the brain surface. The resting membrane potentials (RMPs) of these neurons averaged −69.60±7.64 mV.

### Acoustic responses of AI neurons following electrical stimulation of the MGBv

**Figure 3:**
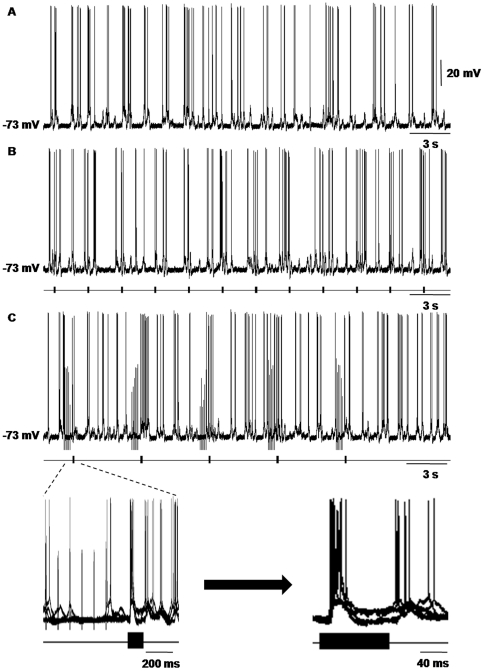
Auditory responses of a cortical neuron after the electrical stimulation of the MGB. **A**) The wave form of spontaneous discharge and oscillation of a single neuron in AI. The frequency of spontaneous discharge was approximately 3 Hz, and the frequency of spontaneous oscillation was approximately 2 Hz. Its RMP was −73 mV, and the baseline of the membrane potential in this neuron was flat and stable. **B**) Auditory neuronal responses to repeated noise-burst stimuli. The discharge rate and frequency of membrane oscillation of the neuron all increased. **C**) Noise-evoked responses of the same AI neuron after the electrical stimulation of the MGBv. The frequency and intensity of train stimulation of the MGBv were 10 Hz and 150 µA, respectively. The left inset figure is the five superimposed traces of the responses of the AI neuron in (B) to the paired stimuli, and the right inset figure is the amplified traces of the left inset figure. It was the clear waveforms of the paired sound-evoked responses in the AI neuron after train stimulation of the MGBv. The waveforms shown as follows were all of this type.

In our results, we recorded the waveforms of spontaneous discharge and oscillation of 40 neurons in AI. We did not begin to perform the experimental protocols until the AI neurons were maintained in stable states. The baselines of the membrane potentials in AI neurons that we recorded were typically flat and steady, and the spontaneous action potentials were overshooting regularly (Fig. 3A). The discharge rates and frequencies of the membrane oscillations of the AI neurons always increased when the animals were given sound stimulations. The noise without the paired ES typically evoked EPSPs with short duration and high amplitude (more than 10 mV) and bursts of spikes on their crests (Fig. 3B). Immediately after the train stimulation, the noise would also evoke an EPSP, but its amplitude and spikes depended on the frequency and strength of the ES (see below). These phenomena were consistent with the results of our previous investigations [19]. The data we analyzed were the average amplitudes of the noise-evoked EPSPs of the same AI neuron before (control groups) and after (experimental groups) the ES of the MGBv. All of the experimental procedures were performed 5 times (Fig. 3C). However, we used 3 to 5 samples in the statistics to eliminate 1 or 2 irregular responses. Thus, all the waveforms that we showed in this article consisted of 3 to 5 superimposed traces of the responses of AI neurons to the paired stimuli.

### Electrical stimulations in the MGBv modulating the auditory responses of AI neurons

**Figure 4:**
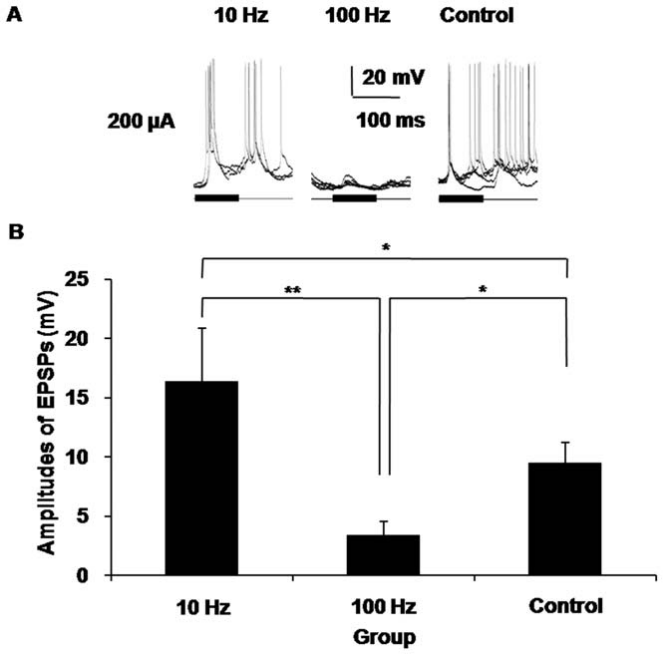
Electrical stimuli in the MGBv modulated the auditory responses of AI neurons. A) High- and low-frequency train electrical stimulation in the MGBv induced different effects in the auditory responses of AI neurons. The amplitudes of the sound-evoked EPSPs increased when the frequency of the train stimuli was 10 Hz and the electric current strength was 200 µA. In contrast, the 100 Hz train electrical stimulation of the MGBv with the same current strength depressed the sound-evoked responses of AI neurons. **B**) The statistical histogram of the average amplitudes of the EPSPs induced by sound after electric stimulation of the MGBv. The data were from the same recordings as in (A). The amplitudes of the noise-evoked EPSPs from the 10 Hz group were higher than those of both the 100 Hz group and the control group. The amplitudes in the 100 Hz group were also lower than those in the control group. (*P<0.05, **P<0.01, Error bars, S.D.).

We used both high- and low-frequency train ES of the MGBv to survey the modulatory effects of electrical stimuli in the MGBv on the auditory responses of AI neurons. In our results, the responses in the control groups, which were the sound-evoked responses of a single AI neuron without stimulation of the MGBv, typically remained stable in waveforms. When the frequency of the train stimuli was 10 Hz and the electric current strength was 200 µA, the average amplitude of the sound-evoked EPSPs increased to 16.31±4.56 mV. This amplitude was significantly higher (*P*<0.05) than that of the control group, which was 9.47±1.72 mV. In contrast, the 100 Hz train ES of the MGBv with the same electric current strength depressed the average amplitude of the sound-evoked EPSPs of the AI neurons to a significantly lower level (*P*<0.05), only 3.36±1.20 mV, relative to that of control group. The diversity of the average amplitude between the 10 Hz group and the 100 Hz group was even higher in statistical significance (*P*<0.01) (Fig. 4). These results indicated that the auditory responses of the AI neurons were indeed modulated by the electrical train stimulation of the MGBv and that the frequencies of the train stimuli played a crucial role in this modulation.

### Stimulus intensity affecting the amplitudes of sound-evoked EPSPs in AI neuron

**Figure 5:**
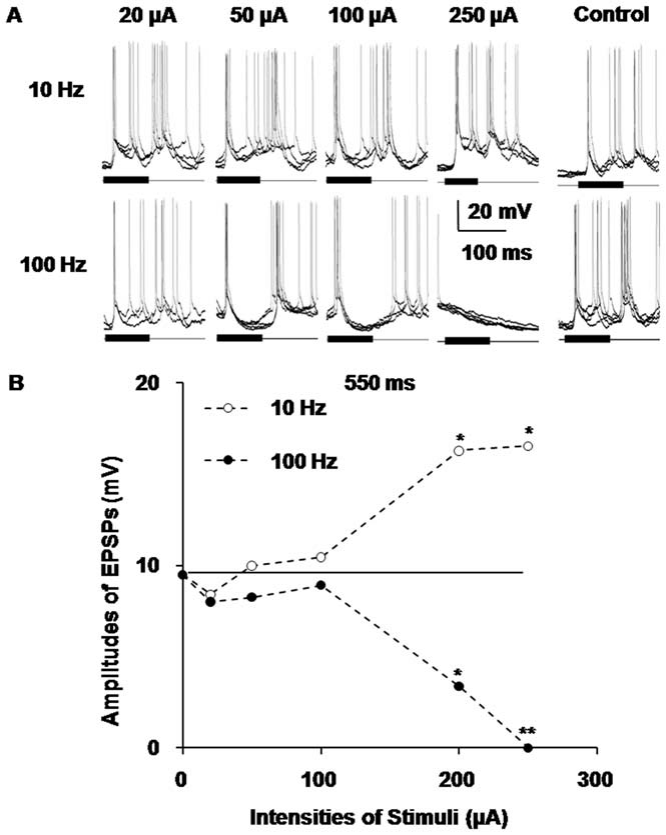
Different responses of AI neurons at varied intensities of train electrical stimulation in the MGBv. **A**) The contribution of stimulus intensity to the amplitudes of sound-evoked EPSPs in AI neurons. Compared with the control group, the amplitudes of the sound-evoked EPSPs were augmented with the increasing of the intensities of the train stimulation when the frequency of the train stimuli was 10 Hz. However, the amplitudes of the EPSPs diminished with the increasing of the intensities of the train stimuli in the 100 Hz group. The 10 Hz train electrical stimulation of the MGBv exerted enhancing effects, and the 100 Hz stimulation exerted weakening effects on the AI neuron's responses to acoustic stimuli. When the intensity was lower than 100 µA, the amplitudes of the EPSPs were either higher or lower than the level of pre-stimuli (control group). **B**) The statistical broken line chart of the average amplitude of the EPSPs evoked by paired sound stimuli after train stimulation of the MGBv at different intensities. The transverse line in the chart was the average amplitudes of the EPSPs evoked only by noises without stimulation of the MGBv, that is, the average amplitudes of the EPSPs in the control group. These data came from the same neuron as in Figure 4. (*P<0.05, **P<0.01).

In our experiments, we further observed different auditory responses of AI neurons at varied intensities of train ES in the MGBv to analyze the contribution of stimulus intensity to the amplitudes of sound-evoked EPSPs in AI neurons. Compared with the control group, the amplitudes of the sound-evoked EPSPs increased as the intensity of the train stimulation increased when the frequency of the train stimuli was 10 Hz. The EPSPs' average amplitudes increased from 9.47 mV to 16.52 mV as the electric current strength increased from 0 µA to 250 µA, and the deviation between the two levels was significant (*P*<0.05). In contrast, in the 100 Hz group, the amplitudes of the EPSPs diminished as the intensities of the train stimuli decreased. In the 100 Hz group, the EPSPs' average amplitudes decreased from 9.47 mV to nearly 0 mV as the electric current strength increased from 0 µA to 250 µA, and the deviation between the two levels was highly significant (*P*<0.01) (Fig. 5). From the statistical broken line chart of the average amplitude of EPSPs, we determined that the magnitude of the effects induced by both high- and low-frequency train stimulations was not statistically significant until the intensities of the stimuli were higher than 150 µA. When the intensity of the current was lower than 100 µA, the amplitudes of the EPSPs were slightly higher or lower than the level of the control group, which ranged from 8 mV to 11 mV (Fig. 5B). These results reaffirmed that electric stimulation of the MGB contributed to the modulation of the auditory responses of AI neurons and that low-frequency train ES of the MGBv exerted enhancing effects, whereas high-frequency ES exerted weakening effects on the auditory responses of AI neurons. Furthermore, they demonstrated that the degree of this modulation depended on the intensities of the train stimuli.

### Different responses of AI neurons at varied intervals between paired stimulations

**Figure 6:**
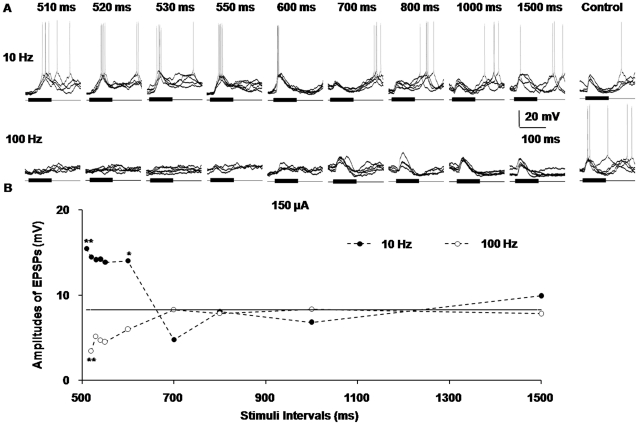
Different responses of AI neurons at varied intervals between paired stimuli. **A**) Five superimposed waveforms of the paired sound-evoked responses in AI neurons after train stimulation of the MGBv at incremental intervals between the paired stimuli. **B**) The statistical broken line chart of the average amplitude of the EPSPs in (A). When the frequency of the train stimuli was 10 Hz, the amplitudes of the sound-evoked EPSPs significantly increased immediately after the train stimulation of the MGBv (compared with the control level), and then decreased to a level lower than the control group when the stimulus interval was extended to 700 ms. Finally, when the stimulus interval was approximately 800 ms, it reached the average amplitude of sound-evoked EPSPs without train stimulation (that is, the level of the control group). In contrast, the 100 Hz train electrical stimulation of the MGBv at first decreased the amplitudes of sound-evoked EPSPs in AI neurons to a significant low level. The amplitudes of the EPSPs then increased to the level of control group. The amplitudes of the EPSPs in both the high- and low-frequency group reached the control level and stabilized at the plateau when the stimulus interval was longer than 800 ms. (*P<0.05, **P<0.01).

In our study, we also observed how the ES of the MGBv induced effects that changed with the extension of the intervals between the paired train electrical and sound stimuli. Compared with the control level of 8.28 mV, when the frequency of the train stimuli was at a low level, the amplitudes of the sound-evoked EPSPs significantly increased to approximately 14–15 mV immediately after the train stimulation of MGBv, which was a highly statistically significant deviation (*P*<0.01) (Fig. 6). Then, the average amplitudes of the EPSPs in the low-frequency group decreased to approximately 5 mV, a level lower than that of the control group, as the stimulus interval extended to 700 ms. Finally, when the stimulus interval was 800 ms, the average amplitudes of the EPSPs reverted to those of the sound-evoked EPSPs without train stimulation (the level of the control group). By contrast, the 100 Hz train ES of the MGBv at first reduced the amplitudes of the sound-evoked EPSPs in AI neurons to approximately 4 mV, a significant low level (*P*<0.05). Then, the average amplitudes of the sound-induced EPSPs increased to the level of the control group as the stimulus interval increased to 700 ms (Fig. 6B). The amplitudes of the EPSPs in both the high- and low-frequency groups reached the average level of the control group and stabilized at the plateau when the stimulus interval was longer than 800 ms (Fig. 6B). These data confirmed the finding that high- and low-frequency stimuli in the MGBv made different contribution to the modulation of acoustic responses of AI neurons. Moreover, they revealed that the degree of this modulation depended on the intervals between the electrical stimulations and their paired sound stimulations.

### Different responses of AI neurons at varied frequencies of train stimulation and stimulus intervals

**Figure 7:**
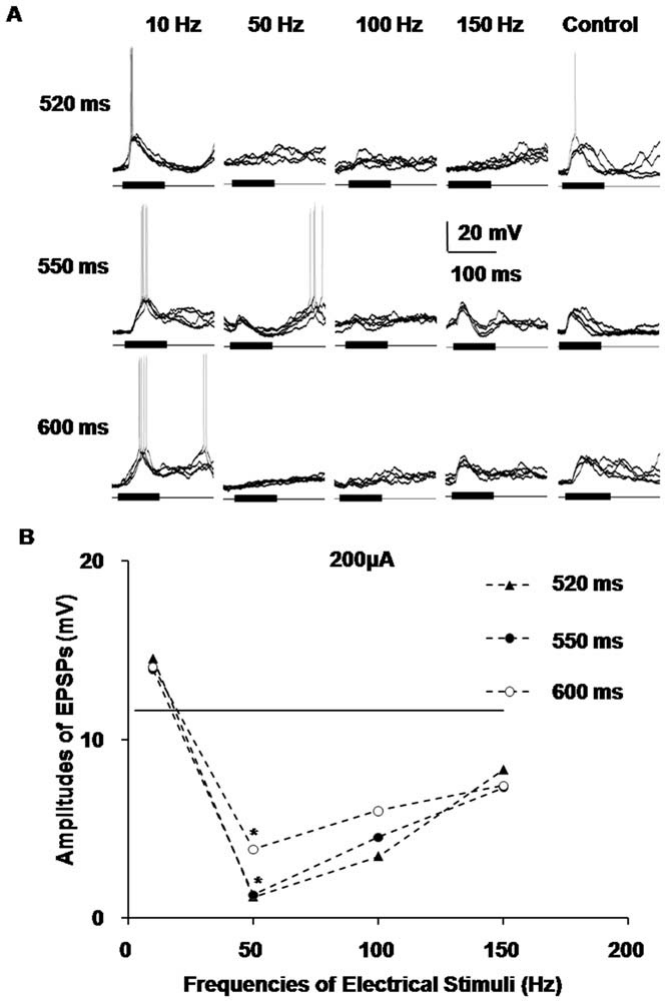
Different responses of AI neurons at varied frequencies and intervals of train stimuli. **A**) Five superimposed traces of the sound-evoked responses in AI neurons after train stimulation of the MGBv at incremental frequencies of electrical stimulation and intervals between the paired stimuli. **B**) The statistical broken line chart of the average amplitudes of the EPSPs in (A). When the stimulus intervals were shorter than 700 ms, the changing tendencies of the different groups of varied stimulus intervals were approximately the same. They all exhibited consistent effects. The amplitudes of the sound-evoked EPSPs of AI neurons increased to higher than those of the control group when the train stimulus frequency of the MGBv was 10 Hz. When the frequencies of the electrical stimuli increased to high levels, including 50 Hz, 100 Hz and 150 Hz, the amplitudes of the EPSPs all decreased to lower than those of the control group. In particular, when the frequency was 50 Hz, the amplitudes of the EPSPs were significantly lower than those of the control group, and might even be close to zero. (*P<0.05).

To enrich and extrapolate the above findings in our research, we extended the experimental protocols to recording the intracellular acoustic responses of AI neurons after train stimulation of the MGBv with incremental intervals between the paired stimuli and incremental frequencies of electrical stimulation. The average amplitudes of EPSPs in the groups with incremental intervals between the paired stimuli, including 520 ms, 550 ms and 600 ms groups, all exhibited consistent effects, either enhancing or depressing effects (Fig. 7). Therefore, we inferred that when the intervals between the paired stimuli were shorter than 700 ms, the changing tendencies of different groups at varied stimulus intervals were approximately the same. Consistent with the aforementioned findings, the amplitudes of the sound-evoked EPSPs of the AI neurons increased to approximately 14 mV, higher than those of the control groups, when the train stimulus frequency of the MGBv was 10 Hz. When the frequencies of the electrical stimuli increased to high levels, including 50 Hz, 100 Hz and 150 Hz, the amplitudes of the EPSPs dipped below those of the control group. In particular, when the frequency was 50 Hz, the amplitudes of the EPSPs decreased to 1.20 mV, 1.30 mV and 3.87 mV, respectively, significantly lower than those of the control group (*P*<0.05) (Fig. 7B). This observation suggested that the effects of the MGB activation were broad-spectrum in electrical frequency to some extent.

## Discussion

### Characteristics of recorded neurons

The average RMPs of the AI neurons that we recorded were approximately −70 mV, which is consistent with that of a normal neuron in the cerebral cortex [20]. It was an uncontroversial standpoint that various acoustic- or non-acoustic-evoked activities of functional neurons in the central nervous system, including spontaneous firing, up and down state, noise- or tone-elicited responses of neurons, etc., depended on the RMP of their neurons [19], [21]–[24]. The healthy states of the recorded neurons were monitored by the overshooting action potentials (Fig. 3A). The discharge rates and frequencies of membrane oscillation of the AI neurons always increased when the animals were given sound stimulations (Fig. 3B). These phenomena were the outcome of the integrations of various excitations and inhibitions elicited by acoustic stimulation of the auditory cortical neurons [25]. They were consistent with the results of our previous investigation and several studies that used intracellular preparations *in vivo* [18], [19]. The calibration and maintenance of the stimulative noise at a fixed intensity of 70 dB SPL provide identical and reliable testing parameters for evoking steady and dependable samples [17], [26], [27].

### The anatomical and functional basis of thalamic activation

Previous studies on thalamocortical transmission demonstrate that ES with different frequencies in the sensory-specific thalamus of the visual, auditory and somatosensory systems can induce either enhanced or depressed effects in their corresponding cortices [13], [15], [28]–[30]. These studies thoroughly revealed the importance of thalamocortical synapses and solid projective basis for either information processing or learning-induced/experience-dependent plasticity of the AC during both early development and adulthood [2]. Additionally, as previously mentioned, there are profuse bidirectional projecting fibers between the AI and MGBv, which makes the neuronal circuitry both compact and complex [31]. Thus, focal ES in the MGBv not only passes to the thalamocortical inputs but also to the intact corticothalamic projections, especially the feedforward inhibitory projections (see below) [32]. The thalamocortical and corticothalamic projections comprise and act as a network for the short-term effects on the AC induced by thalamic activation [33].

Furthermore, the thalamocortical projection is organized with highly convergent fibrous bundles [34]. Therefore, it is possible that the neurons that we activated via ES in the MGBv are not the same neurons that convey the sound-evoked information in the thalamus. Due to the limited spread of ES and the different activating states of thalamic neurons, the changes in the amplitudes of EPSPs do not appear in lower intensity groups (<100 µA) [35]. The modulatory effects of cortical responses after ES appeared only in the 100 µA group and could be more effective in higher intensity groups (>100 µA) (Fig. 5A).

### Low-frequency activation of MGBv enhances the auditory responses of AI neurons

The low-frequency thalamic activation resulted in facilitatory effects on the amplitudes of sound-evoked EPSPs of AI neurons. This type of low-frequency electrical stimulation of the thalamus, which was the low-pass temporal filter of the afferent information to the cortex, plays important roles in cortical gain control as well as the balance between excitation and inhibition of AI [36]–[39]. The same acoustic stimulation induces stronger responses after low-frequency thalamic activation than the responses without train stimulation. This finding indicates that the low-frequency electric stimulation of the thalamus leached the inputs of other sound information and reduced the background noise of AI neurons. As a result, the responses of the neurons in the AI to ascending auditory signals are reinforced. The function of the low-pass temporal filter took place within 200 ms after the onset of thalamic electric stimulation. This filtration effect is the reason why the amplitudes of sound-evoked EPSPs increased to higher levels than that of pre-stimuli when the stimuli intervals were shorter than 700 ms.

Previous studies had showed that low-frequency train stimulation could enhance the processing of neural signals by acting the feedforward inhibitory (corticothalamic) neurons to some extent, which diminished the activities of the thalamocortical projections and increased the amplitudes of the post-synaptic responses at the same time [33], [40]. The reduction of the activities of the thalamocortical cells is mediated through an N-methyl-D-aspartic acid (NMDA) receptor-dependent mechanism in the thalamus. The introduction of an NMDA receptor antagonist into the thalamus can directly enhance the efficacy of thalamocortical transmission [41]. Thus, we speculate that the effects of low-frequency train stimulation of the MGBv are likely to be based on the N-methyl-D-aspartic acid (NMDA) receptors [8], [42]. Of course, the exact reason relies on further investigations.

### High-frequency activation of the MGBv depresses the auditory responses of AI neurons

The high-frequency thalamic activation obviously exerts an inhibitory effect on the amplitudes of sound-evoked EPSPs in AI neurons. The activation of the MGBv with high-frequency electric stimuli might elicit different consequences. On the one hand, it is well known that the inhibitory GABAergic neurons constitute only a negligible proportion (<1%) of the total neurons of the MGBv, so the inhibitory effect might not be simply caused by inhibitory afferents of thalamocortical projections directly [43]. However, the MGBv sends fibers to the interneurons in AI with widespread axonal projections and contacts with each other to trigger an inhibitory neural network. This inhibitory network constructs the foundation of the inhibitory circuits in the whole AI and induces an immense release of the inhibitory neurotransmitter GABA (gamma-aminobutyric acid) [44]. On the other hand, prior investigations and our results reveal that the inhibitory effect induced by high-frequency ES lasts for several hundreds of milliseconds. This duration is presumably due, at least in part, to the vast release of modulatory neurotransmitters, including NMDA (N-methyl-D-aspartic acid), to the cortical layers. Moreover, both high release probability and multiple release sites of thalamocortical axons are required in this instantaneous release elicited by tetanus stimulation of the MGBv [8].

Therefore, according to the former and our current studies, we hypothesize that the inhibitory effects depend, to a great extent, on the rapid depletion of the readily releasable pool of vesicles in the pre-synaptic terminals, which leads to a lack of excitatory transmitters to drive the postsynaptic neurons [12]. Both of the two aspects are most likely potential pathways for the depression of auditory responses in AI neurons by high-frequency thalamic activation and are the functional basis of the thalamocortical contributions to information processing in AI as well. However, what these inhibitory phenomena are attributed to should be clarified via pharmacological identification in the future. These findings provide a new clue for investigations of the mechanism of the neural coding of temporal information [45], [46].

### Conclusion

It has been shown that a train of classical high-frequency electrical-pulse stimulations of the thalamus induces gradually enhanced waveforms in the cortex, whereas low-frequency electrical-pulse stimulations induce suppressed waveforms [47]. In addition, the recent sensory experiences can affect the successive responses of cortical neurons to the afferent sensory stimulations [14], [48]–[50]. However, we do not know what happens to the cortical processing of ascending sensory information after the electrical stimulation of the thalamus, that is, how the cortical response to natural stimuli is changed by the electrical stimulation of the thalamus. Our results revealed that, although evoked by the same sound, the amplitudes of the EPSPs of a single AI neuron increased after the low-frequency stimulation of the MGB, whereas high-frequency stimulation depressed the auditory responses. This research constitutes a further step toward demonstrating the intrinsic mechanism of the contribution of the thalamocortical system to the responses of the AC and to cortical signal processing.

## Materials and Methods

Thirty-six adult female Sprague-Dawley rats with body weights ranging from 230 to 250 g served as subjects in the present study. All of the experimental protocols and procedures were designed to conform to Ethics in the Care and Use of Laboratory Animals of China and were approved by the Animal Care and Use Committee of the Third Military Medical University.

### Animal preparation

The animals were anesthetized with urethane (ethyl carbamate, 1.5 g/kg, intraperitoneal injection) during surgeries and throughout the experimental processes. The anesthetic status was examined approximately every hour by pinching the animal's forepaw with forceps. If necessary, an additional dose of urethane (0.2 g/kg, i.p.) was administered to maintain suppression of the forepaw withdrawal reflex. We performed tracheotomy and endotracheal intubation and vacuumed off any potentially fatal lung secretions to maintain smooth breathing throughout the experiments. The rat's capills in the head were cut down and the scalp was incised at the midline. The subcutaneous tissue and muscle in the corona capitis and right temple side were removed to expose the roof and right skull. Then, the animal's head was secured in a custom-made head holder by solidly clamping between the palate and nasal/frontal bones. The head clamp was adjusted to align the bregma and λ points of the skull in a single horizontal plane. Each rat's back was suspended at approximately the same height as the level of the neck to minimize the mechanical fluctuation of the cortex caused by the breathing [51].

The AI intracellular recording sites, which had been determined by extracellular recordings in advance, were precisely measured and marked on the temporal skull according to the stereotaxic map of the auditory and surrounding areas of cortex derived from the rat atlas (2.7 to 5.8 mm posterior to the bregma and 3.1 to 5.4 mm ventral to the bregma) [52]. As the right temporal bone was scraped to thin by an abrazine aiguille, the characteristic blood vessel pattern that frames the AI was revealed gradually [53]. A hole measuring less than 1 mm in diameter for the placement of the recording electrode was made approximately at the center of the marked area, and the dura/pia-maters were carefully removed with a sharp needle [51]. Warmed low-melting-temperature paraffin was dropped on the exposed AC periodically to keep it moist. Another hole, measuring 1 mm in diameter (5.0 to 6.0 mm posterior to the bregma, 3.0 to 4.0 mm lateral to the midline), was drilled with a dental electrodrill in the roof of the skull for the corresponding placement of an electrode to the right MGBv of the thalamus [52]. The body temperature was maintained at 37°C by a feedback-controlled heating pad beneath the rat's body. The power for the heating pad was supplied by a direct current accumulator battery. After surgery, the animal was placed in a double-walled, electrically shielded and soundproof chamber for the following electrophysiological experiments.

### Acoustic stimulus

The acoustic stimuli were a series of white noise or pure tone bursts with 5 ms rise/decay times and 100 ms duration at different intervals. The acoustic signals were digitally synthesized with SigGen software and played by BrainWare software (Tucker-Davis Tech., FL, USA), which also allowed the selection of the frequency and the attenuation of the acoustic signals. Digital signals were converted into analogue signals through the Real-time Processor (RP2), whose output amplitude of sinusoidal waves was set at 20 V peak-to-peak without attenuation during calibration. The signals were then fed to a computer-controlled attenuator (PA5) and presented by an electrostatic speaker through an electrostatic speaker driver. The speaker, placed 45° to the left of and 10 cm away from the rat's left ear, was calibrated at the position of the animal's left ear with a condenser microphone (Model 377A01, PCB PiezotronicsINC, USA) and a microphone preamplifier (Model 426B03, PCB PiezotronicsINC, USA) before the experiment. The output frequency of the tone burst ranged from 3 to 40 kHz, consistent with the frequency RFs of the adult rat, and was expressed as decibel sound pressure level (dB SPL). Tone bursts with various frequencies and intensities were delivered either manually or automatically via BrainWare software. The testing noise was fixed at the intensity of 70 dB.

To measure the extracellular excitatory response thresholds across different frequencies or to determine the frequency/threshold-tuning curves of auditory neurons in AI and the MGBv, a computer-controlled frequency/amplitude scanning (frequency scan plus amplitude scan, FA-scan) were presented to the animal for 5 times. In each FA-scan, the tone frequency (varied from 3 to 45 kHz in increments of 1 kHz) and amplitude (varied from −10 to −70 dB with increments of 5 dB) were randomly arranged by the BrainWare software. Therefore, it consisted of 559 frequency/amplitude sets. The interval between tone bursts was 500 ms.

### Location and ES in the MGBv

A parylene-insulated tungsten electrode of ∼2 MΩ tip impedance was perpendicularly advanced to the stereotaxic coordinates position of the right MGBv (5.5 mm posterior to the bregma, 3.5 mm lateral to the midline, and about 5.0 mm below the brain surface) [52], to derive the BF, MT, and frequency/threshold-tuning curves of the MGBv neurons [15]. This electrode was initially connected to the preamplifier of the recording system (Fig. 1A). After the electrode was advanced to approximately 4 mm below the pia, a pure tone with manual alteration of frequencies and amplitudes was continuously delivered at a rate of 1 Hz to facilitate the search for neurons responding to tone stimulus during the electrode penetration. Bioelectrical signals from the electrode were fed to an RA16PA multichannel preamplifier and were filtered with a bandpass of 0.3–10 kHz and amplified 10,000 times by the RA16 Medusa Base Station (Tucker-Davis Tech., FL, USA). Next, the processed output signals were transmitted to an oscilloscope for observation and were stored based on eight parameters of the action potential waveform including peak, valley, spike height, spike width, peak time, valley time, and two selected voltage values, using BrainWare data acquisition software (Tucker-Davis Tech., FL, USA). Once a neuron responding to a tone stimulus was observed, the electrode was continuously lowered until there were no recorded auditory responses, which represented the ventral boundary of the MGB. Afterwards, the electrode was slowly withdrawn by 50–100 µm to record the responses of MGB neurons to the FA-scan. If the recorded tuning curve was not sharp at a certain frequency, the electrode was withdrawn to the brain surface and another electrode penetration near the anterior sites was performed, repeatedly if necessary, until sharply tuned neurons were recorded. This method was established in previous studies on mice [15], [54], [55], and it ensured that the electrode tip was within the MGBv, as supported by the histological examination of the lesion mark (Fig. 2B).

The electrode was then carefully fixed in the rat's skull with cyanoacrylate glue and dental cement to maintain its position, but its initial role was changed to serve as a stimulating electrode to provide ES in the MGBv by switching its connection from the recording system to a Grass S88 stimulator (AstroMedical, West Warwick, RI, USA) and an A360 constant-current isolator (World Precision Instruments, FL, USA). The negative pole of the isolator was connected to the stimulating electrode, and the positive pole was connected to the rat's scalp through a metal clip.

The ES was performed in patterns of train pulse stimulation (TPS). The TPS consisted of a train of identical single-pulse stimulations at different frequencies. Each single-pulse stimulation was a monophasic and negative constant current square wave ES lasting for 0.2 ms. The level of the stimulation current was manually set within a range of 0–250 µA via the isolator [24], [56], [57]. Each stimulus with certain parameters was repeated 5 times. The interval between every two stimuli, controlled either manually or automatically via the stimulator, was longer than 5 s during TPS to diminish the interaction between consecutive stimuli (Fig. 1B). Each pair of electric and sound stimuli was performed for 5 times.

### Intracellular recording in the AI

We used extracellular recording for the general features of the right AI. It was determined by testing the RFs of AI neurons through another tungsten electrode, in a manner similar to the above recording in the MGBv [58]. As the electrode was perpendicularly advanced under the cortical surface in the hole drilled for AI recording, a pure tone with manual variation of frequencies and amplitudes was continuously delivered until a neuron responding to a tone stimulus was observed. The responses of the AI neurons were commonly derived when the electrode tip was 300–400 µm below the brain surface [59].

The tungsten electrode was removed after mapping of the AI, and a sharp glass pipette (filled with 1.0 M potassium acetate, tip impedance: 80–100 MΩ) for intracellular recording was immediately penetrated vertically to the AI surface by a stepping motor (Narishige, Tokyo, Japan) at a location just adjacent to the penetrating point made by the tungsten electrode. Electrical signals were fed to a wide-band active probe electrometer (intra-767, World Precision Instrument, USA) by a silver wire, filtered with 0–6000 Hz and amplified 10,000 times. A high-impedance amplifier with active bridge circuitry was used to record the membrane potential and inject current into the neurons if necessary. The level of the direct current of the recording electrode was checked frequently and set to zero during the experiments. Cortical responses resulting from thalamic stimulation were recorded under different levels of membrane polarization, obtained by current injection through the electrode (from −1 to +1 nA steady current) [60]. The reference electrode was also connected to the rat's scalp through a metal clip. When the glass electrode tip was approximately 200 µm below the AI surface, it was slowly advanced at 1 or 2 µm per step. Following every 2 to 3 steps of penetration, the ‘Tickle’ button on the control panel of intra-767 electrometer was pressed briefly to cause a click of electrical oscillation at the electrode tip; this operation facilitated the penetration of the tip into a living cell. The level of the direct current after a successful recording was used to compensate for the membrane potential of certain neurons, especially for those with a long recording time. Additionally, the recording depth of the electrode tip was limited from 200 to 1300 µm below the brain surface. The output of the intra-767 electrometer was digitized at a sampling rate >20 kHz with a digital analog converter micro1401mkII (Cambridge Electronic Design, Cambridge, UK), monitored and saved via Spike2 software (Cambridge Electronic Design, Cambridge, UK).

### Anatomic localization and histology

After all the electrophysiological experiments were completed, an electrical current of 400 µA and 30 s duration was applied to the site of ES in the MGBv, which created a small lesion at the recording site (Fig. 2B). The animal was overdosed with urethane (2 g/kg, i.p.) and perfused through the heart with 0.1 M phosphate-buffered saline (PBS, pH 7.4) followed by a mixture of 4% polyoxymethylene in phosphate buffer (PB, pH 7.4). The brain was carefully removed from the skull and post-fixed with 4% polyoxymethylene/30% sucrose in PB for 24 to 48 h at 4°C for cryoprotection. The brain was then sectioned in the coronal plane (50 µm in thickness) with a freezing microtome and collected in 0.05 M PB. The brain slices were tiled on the microscope slides, which had been treated with polylysine, and air-dried overnight at room temperature. The tissue was stained for Nissl substance with toluidine blue for the visualization of the electrolytic lesion points that manifested the actual loci of stimulation under the microscope (Fig. 2B).

Following 30 s of rinsing in distilled water (H_2_O), the slides were immersed in 75%, 85%, 95%, 85%, and 75% alcohol in sequence for 10 min each, to be degreased, and then re-immersed in H_2_O for 10 min. Subsequently, the slides were immersed in 1% toluidine blue stain aqueous solution (1 g toluidine blue per 100 ml H_2_O) that was preheated to 50°C and placed in an incubator at 56°C for 20 min to be stained. Next, the tissue was rinsed in H_2_O, followed by 1 min of reacting in 70% alcohol. Thereafter, the tissue was steeped in 95% alcohol to accelerate the differentiation of the toluidine blue stain until the Nissl body was definite under the microscope. Dehydration with anhydrous alcohol was performed immediately, followed by clearing in dimethyl benzene. The brain slices on the microscope slides were cover-slipped and mounted with neutral gum.

### Data acquisition and analysis

The intracellular recording data were stored in a computer using the Spike2 software and analyzed off-line. Once penetrating the membrane of a cell, the electrode detected a sharp drop in membrane potential. Neurons showing a RMP less than −50 mV, or lasting for less than 20 min in recording duration, commonly indicated unhealthy cells and were excluded in our present study. Each sample in the analysis was averaged from 3–5 repeated test stimuli.

The numerical results are expressed as the means ± standard deviation (S.D.). A *t*-test for independent samples was used to examine the differences between diverse sets of postsynaptic potentials (PSPs) or changes in membrane potential evoked by acoustic stimuli after ES of the MGBv, using *P*<0.05 (*) and *P*<0.01 (**) as the criterion for statistically significant deviation.
